# Total hip arthroplasty to treat acetabular protrusions secondary to rheumatoid arthritis

**DOI:** 10.1186/s13018-018-0809-y

**Published:** 2018-04-19

**Authors:** Ping Zhen, Xusheng Li, Shenghu Zhou, Hao Lu, Hui Chen, Jun Liu

**Affiliations:** 1 Department of Orthopedics, The Second Affiliated Hospital of Lanzhou University, Lanzhou, 730030 Gansu People’s Republic of China; 2 Department of Orthopaedics, Lanzhou General Hospital of PLA, No. 333 South Binhe Road, Lanzhou, 730050 Gansu People’s Republic of China

**Keywords:** Acetabular protrusion, Rheumatoid arthritis, Autogenous bone transplantation, Hip arthroplasty, Reconstruction

## Abstract

**Background:**

The treatment of acetabular protrusions during total hip arthroplasty of patients with rheumatoid arthritis is difficult. A lack of bone stock, deficient medial cup support, and medialization of the joint center in those with protrusio acetabuli must be addressed during acetabular reconstruction. The purpose of this study was to assess the short-term clinical results of total hip arthroplasty in patients with severe acetabular protrusions secondary to rheumatoid arthritis.

**Methods:**

From January 2011 to November 2014, 18 patients (20 hips) with severe acetabular protrusions secondary to rheumatoid arthritis underwent total hip arthroplasties using a non-cement impaction and auto-bone-grafting method with resection of the femoral head to treat the acetabular protrusion. The Harris hip scoring system was used to evaluate hip function during follow-up; X-rays were taken to assess the extent of prosthesis loosening and bone graft healing.

**Results:**

The operation time ranged from 55 to 131 min, averaging 89.5 ± 8.1 min. The blood loss was 165–480 mL (295 ± 10.9 mL). No blood vessel or nerve damage and no acetabular or femoral fracture occurred. The follow-up duration was 4.5 ± 1.7 years. Postoperative X-rays revealed autologous bone graft/acetabular fusion at 4.5 months post-surgery. The Harris hip scores increased significantly, from 55.3 ± 9.5 to 92.2 ± 12.7, after the operation (*P* < 0.01). The distance from the center of the femoral head to Kohler’s line increased from 19.87 ± 3.9 mm to 21.5 ± 3.5 mm after the operation (*P* < 0.01). During follow-up, no hip acetabular prosthesis loosening was evident.

**Conclusions:**

For patients with protrusio acetabuli secondary to rheumatoid arthritis, the use of a cementless, trabecular, metal modular cup allowing peripheral press fitting and restoration of bone stock via impacted autologous bone grafting are both technically straightforward and appear to yield satisfactory short-term results.

## Background

Rheumatoid arthritis (RA) is the most common inflammatory joint disease [1]; lesions involving the hips, knees, and other large joints can progress to secondary joint deformity and dysfunction [2]. In the late phase of hip disease, the deformed femoral head causes irregular abrasion of the acetabulum, secondarily shifting the femoral head inward and in turn triggering varying degrees of the acetabular protrusion, the incidence of which is about 5% [3]. Notably, acetabular protrusion developing secondary to RA differs from the typical lesions, being associated with thinner ventral acetabular bone, a weaker acetabular rim, a lack of bone stock, stronger local osteoporosis, and a more irregularly shaped acetabulum. Total hip arthroplasty is very difficult to perform in such patients because the initial stability of an acetabular prosthesis cannot be guaranteed [2–5].

## Methods

This study was approved by the Medical Ethics Committee of the Lanzhou General Hospital of the Peoples’ Liberation Army. Informed consent was obtained from all individuals. The study was performed in accordance with the Declaration of Helsinki, as revised in 2008. All patients provided written informed consent prior to participation.

### Inclusion and exclusion criteria

The inclusion criteria were (1) adults diagnosed with RA complicated by hip joint involvement as defined by the Chinese Medical Rheumatology Association (2010) [6], (2) pelvic, anteroposterior radiographic evidence that the acetabular site had moved over Kohler’s line (or Nelaton’s line) in patients with acetabular protrusions, and (3) the availability of complete medical records and follow-up information. The exclusion criteria were (1) RA in combination with any other disease rendering surgery impossible and (2) acetabular protrusion developing secondary to hip joint trauma or another cause.

### Patient information

Between January 2011 and November 2014, 18 patients (20 hips) with protrusio acetabuli secondary to RA were treated via total hip arthroplasty at our institution. There were 6 males (6 hips) and 12 females (14 hips). Patients’ age ranged from 37.0 to 68.5 years (mean = 45.8 ± 8.3 years). Two patients had bilateral lesions and seven left and nine right lesions nine left and right lesions, respectively. The principal clinical manifestations were hip pain during standing and walking and limitation of joint movement. Severe (outreach angle < 25°) and mild (outreach angle > 25°) abduction limitations were evident in 12 cases (14 hips) and 6 cases (6 hips), respectively. In addition, nine cases (11 hips) exhibited muscle strength of grade IV, and nine cases (9 hips) had muscle strength of grade III according to MRC muscle grading. The Trendelenburg sign was always positive. The duration of RA-related hip disease ranged from 8 to 17 years (mean = 9.2 ± 1.3 years).

#### Imaging

Routine X-ray and computed tomography (CT) scans of the hip were obtained. X-rays of the anteroposterior pelvis aided in the diagnosis of the acetabular protrusion, which was evaluated by the relative positions of the acetabular wall and Kohler’s line. The extent of the protrusion in the 18 patients (20 hips) ranged from 6 to 17 mm (mean = 12.17 ± 4.2 mm). Using the Sotello-Garza and Charnley classification [7], 15 cases (17 hips) were of type II (protrusion 6 to 15 mm), and 3 cases (3 hips) were type III (> 15 mm); we encountered no type I (1–5 mm) case.

### Surgical technique

Total hip arthroplasty was performed under general or spinal anesthesia. In all cases, the posterolateral approach was used, with the patient placed on the contralateral side. Commencing 1 cm posterior to the tip of the greater trochanter, a straight skin incision approximately 12 cm in length was created. The gluteus medius was retracted, and the short extrarotators were isolated and detached. The insertion of the quadratus was spared if possible. Because the femoral head protruded intrapelvically, it was difficult to dislocate the femoral head in surgery. In those with severe protrusions, the femoral head and neck were completely trapped in the acetabulum, and common osteotomy of the femoral neck was difficult to perform. In such patients, part of the edge of the femoral head and part of the neck lying inside the acetabulum were first removed using a drill or a narrow bone knife. Exploiting lower limb traction and abduction, the femoral neck was then partially exposed to facilitate osteotomy. As the femoral head had retracted and adhered inside the protrusion into the acetabulum, it was difficult to remove the head intact; thus, we removed the pieces created by breakage. The acetabular preparation was performed in two stages [8]: that of the periphery and then that of the medial floor. Unlike the common case in standard reaming, we did not start with the smallest reamers with the aim of preparing the floor first. Instead, the peripheral cartilage was reamed down at first, albeit preserving the subchondral bone using larger reamers, to one or two sizes smaller than the final cup size templated. The aim was to achieve firm contact between the peripheries of the cup and as wide as a band of the acetabular rim as possible. The acetabular rim should be reamed shallow circumferentially only inside the rim, with the decided cup abduction angle to obtain a perfect shape of the rim because an acetabular rim in RA tends to become thin. Second, in preparation of the medial floor, we gently resected the synovial tissue and soft tissue in geodes in the acetabulum and then only exposed bleeding subchondral bone with drilling multiple small holes on the acetabular wall using a Kirschner wire wherever the subchondral bone appeared to be very sclerotic. The medial reaming was therefore not performed because it can lead to penetration of the thin medial wall. The cup should be in contact with at least 50% of good quality acetabular bone for adequate stability of the cup to facilitate bone ingrowth into the shell and provide sufficient mechanical support to the underlying graft. With regard to the preparation methods of grafting bone, the resected femoral head was fined using a bone mill with rongeurs not "holes" holes 5 mm in diameter in 20 hips. In the eight cases of severe protrusion or remarkable destruction and resorption of the acetabulum, we added extra bone that is supplied from the pelvic wing. For the methods of impaction bone grafting, the trial head of the hemiarthroplasty prosthesis is used. A trial head with a size smaller than the diameter of the acetabulum is used for the impaction of the grafting bone until rigid compression of the bone is obtained. After the impaction technique is employed, the cementless acetabular component is inserted and fixed with supplemental screws. All patients were allowed to transfer from the bed to the wheelchair on the first postoperative day. If satisfactory cup rim fixation was evident during surgery, full weight bearing was also allowed on the fifth day not first day first day after surgery.

**Fig. 1:**
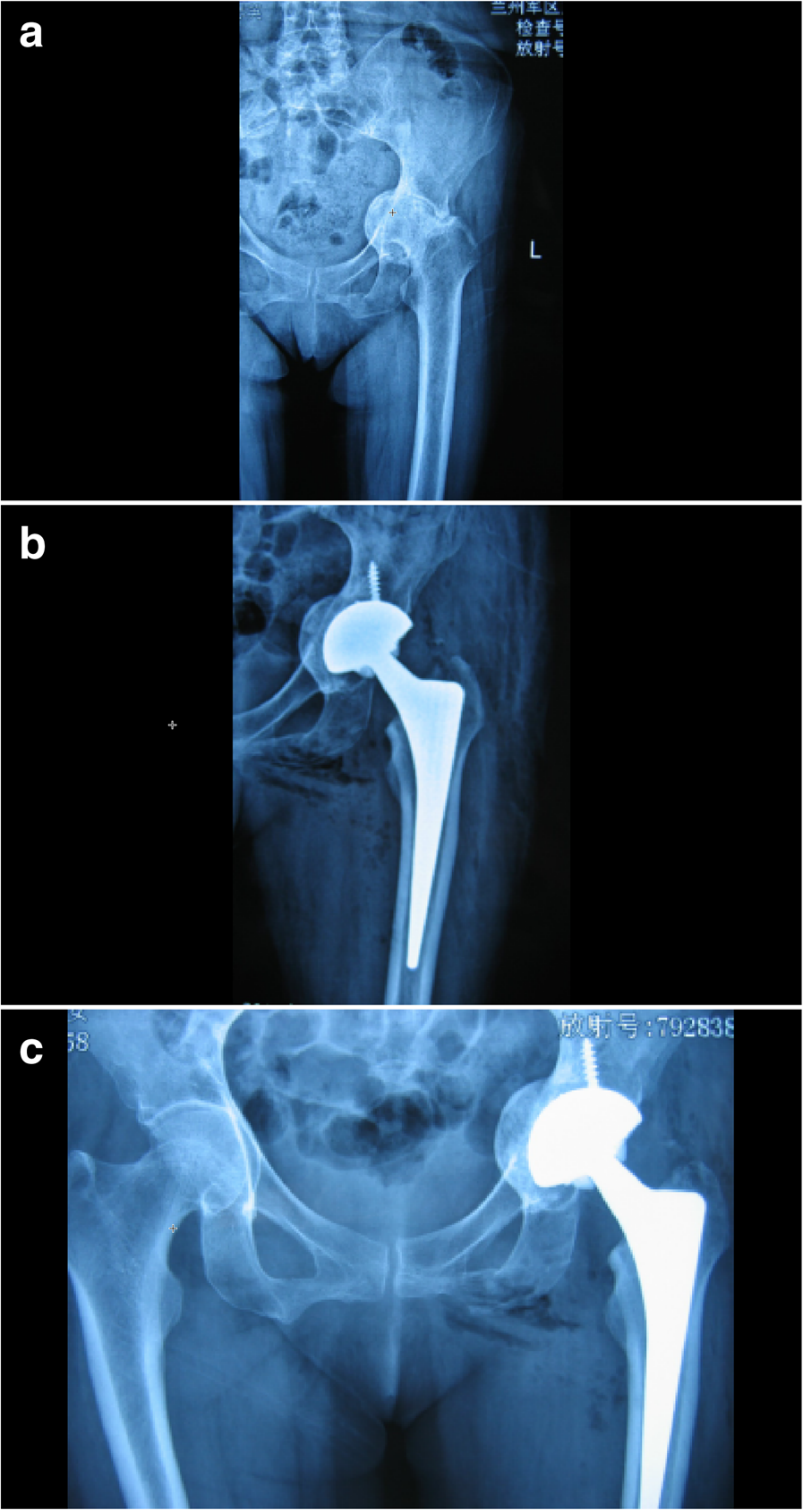
**a** Left hip exhibiting rheumatoid arthritis (RA) with an acetabular protrusion. The femoral head protruded inward over Nelaton’s line. The disease was of Sotello-Garza and Charnley type II. **b** Total hip arthroplasty (featuring placement of a porous tantalum acetabular cup; Zimmer) accompanied by acetabular reconstruction with autologous bone. An X-ray taken immediately after surgery revealed that the hip joint rotational center had returned to the normal location. The initial acetabular cup stability was good. **c** At the 5-year follow-up, an X-ray revealed complete bone graft healing, without bone resorption or acetabular loosening

**Fig. 2:**
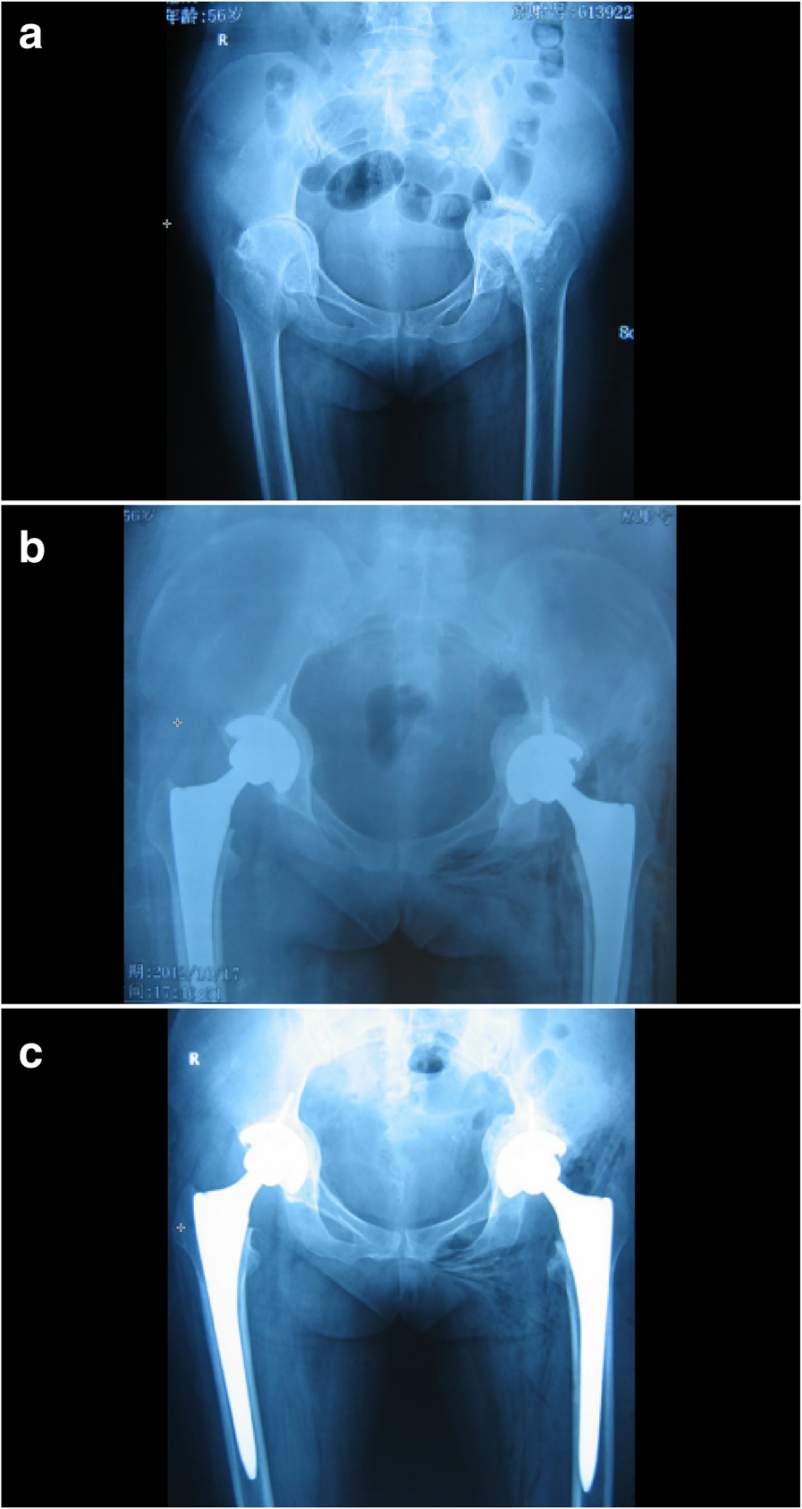
**a** A bilateral acetabular protrusion developing secondary to RA; the bilateral femoral head protruded inward over Nelaton’s line. **b** Single-stage not session session bilateral total hip arthroplasty (with the placement of a titanium-coated, biopsy acetabular cup; Smith & Nephew, USA) accompanied by acetabular reconstruction using autologous bone. An X-ray taken immediately after surgery showed that the rotational center of the hip joint had returned to the normal anatomical location. The initial stability of the acetabular cup was good. **c** At the 4-year follow-up, the X-ray revealed complete healing of the bilateral bone graft, without bone resorption, an acetabular protrusion, or loosening

Given the thin acetabular rim and the fact that bone grafts were placed on the poor quality of acetabular bed, the acetabular components of the total hip arthroplasty that were used in this series were as follows: porous tantalum acetabular cups (TM cups; Zimmer, USA) were placed in 16 patients (16 hips) (Fig. 1), and sintered, three-dimensional, asymmetric, titanium, porous coated cups (R3 cups; Smith & Nephew, USA) were placed in 2 patients (4 hips) (Fig. 2). The weight-bearing interfaces of eight cases (8 hips) received the fourth generation ceramic-ceramic implantations; ten cases (12 hips) received high-level cross-linked polyethylene-lined ceramic implantations.

### Postoperative follow-up and evaluation

The Harris scoring system [9] was used to assess hip function before and after surgery. Radiographs of the operated hips were obtained immediately after surgery, and at 6 weeks, 3 months, and 6 months, and annually thereafter. The radiographs were carefully assessed by the senior surgeon and an independent observer. We measured the central, postoperative, hip rotation recovery on anteroposterior radiographs of the pelvis; this was reflected by (1) the vertical distance of the new hip center from the line of the ischial tubercle connection and (2) the horizontal distance of the hip center from Kohler’s line [10].

The immediate postoperative and follow-up radiographs were compared to assess bone regeneration. Bone graft healing was evidenced by the development of continuous trabecular bone throughout the graft and the host bone interface, as revealed by X-rays. Loosening of the acetabular prosthesis was reflected by a shift (horizontal or vertical) of the acetabular cup of > 5 mm or the presence of transparent lines around the prosthesis [11]. Bone absorption within the acetabular bone graft was determined by the Gerber method [12], a transparent line around < 33% of the graft reflected mild reabsorption, while a line around 33–50% of the graft moderate indicated reabsorption and a line around > 50% of the graft severe reabsorption.

### Statistical analysis

SPSS software (ver. 19.0; SPSS Inc., USA) was used for all data analyses. The data are presented as means ± standard deviation (SD). The difference between the Harris hip score and the hip rotational center was evaluated using a paired *t* test. A *P* value < 0.05 was considered statistically significant.

## Results

### Operational parameters

The operation time ranged from 55 to 131 min (mean = 89.5 ± 8.1 min). The blood loss ranged from 165 to 480 mL (mean = 295 ± 10.9 mL). No blood vessel or nerve damage occurred, and no acetabular or femoral fracture was noted.

### Functional outcomes

All patients were followed up for 2.5 to 6 years. The mean preoperative Harris hip score was 55.3 ± 9.5, which improved significantly, to 92.2 ± 12.7, at a mean follow-up of 4.5 years (*t* = 22.81, *P* < 0.01). Hip flexion and extension increased from a preoperative value of 41.5° ± 6.7° to 102.3° ± 14.5° at the final follow-up. Patients could walk without any aids, including up and down stairs, and could put on shoes and socks independently; subjective patient satisfaction was good (Table 1).

**Table 1:** Patient demographics

Demographics	Data
Gender	6 males (6 hips), 12 females (14 hips)
Mean age	45.8 ± 8.3 years (range 37.0 to 68.5)
Course of disease	8~17 years (mean 9.2 ± 1.3 years)
Acetabular type	Type I protrusion (1~5 mm), 0 case; type II protrusion (6~15 mm), 15 cases (17 hips); type III protrusion (> 15 mm), 3 cases (3 hips)
Outreach angle	Extension angle < 25°, 12 cases (14 hips); mild extension limitation (extension angle > 25°), 6 cases (6 hips)
Abductor muscle strength classification	Grade IV, 9 cases (11 hips); grade III, 9 cases (9 hips)
Operation time	55~131 min, 89.5 ± 8.1 min
Blood loss	165~480 mL, 295 ± 10.9 mL
Preoperative Harris score	55.3 ± 9.5 (40~65)
Postoperative Harris score	92.2 ± 12.7(89~95)
Preoperative hip flexion and extension activity	41.5° ± 6.7°
Hip flexion and extension at the last follow-up	102.3° ± 14.5°
Distance between the preoperative femoral head center and the ischial tubercle connection	77.55 ± 12.3 mm
Distance from the femoral head to the ischial tubercle at final follow-up	72.83 ± 11.1 mm
Distance from the femoral head center to the Kohler line	19.87 ± 3.9 mm
Distance from the femoral head center to the Kohler line	21.5 ± 3.5 mm
Follow-up time	2.5~6, 4.5 ± 1.7 years

### Radiographic findings

The immediate, postoperative, anteroposterior pelvic radiographs showed that the acetabular cups and femoral stem prostheses were correctly positioned in all cases (20 hips). The mean acetabular inclination angle was 43.5° (range 40°–50°). In all cases, radiographs showed that the impacted morcellized bone grafts became incorporated into the surrounding bone (Fig. 1). Follow-up radiographs showed that the bone grafts united within 4 to 6 months (mean = 5.5 months) after surgery, and continuous trabecular bone that grew through the junction of the host bone and the bone graft was apparent. All acetabular components were stable at the last follow-up (Fig. 2), and no perceptible positional change was noted. At the final follow-up, no radiolucency or sign of bone graft absorption was apparent at the periphery of the cup. No component migrated superiorly or medially, and we found no evidence of recurrence of protrusio acetabuli in any case. The distance from the femoral head center to Kohler’s line increased from 19.87 ± 3.9 mm preoperatively to 21.5 ± 3.5 mm postoperatively (*t* = 2.312, *P* < 0.01); the distance between the femoral head center and the ischial tubercle connection decreased significantly from 77.55 ± 12.3 to 72.83 ± 11.1 mm postoperatively (*t* = 4.221, *P* < 0.01). Thus, the new hip rotation center migrated from a superior and medial position to a lateral and inferior position, which is normal.

## Discussion

Intrapelvic protrusion of the femoral head (protrusio acetabuli) may be either primary or secondary. Secondary protrusio acetabuli is associated with various disease states, including hip arthritis, trauma, or metabolic diseases, as well as infection, RA, and Paget’s disease [1, 3–5]. RA and ankylosing spondylitis are the most common conditions encountered, and the use of corticosteroids is an important risk factor for acetabular retraction [1, 2]. Notably, in contrast to other secondary lesions, hip joints affected by RA often exhibit synovial congestion, edema, and erosion of the articular cartilage surface; the femoral head is commonly destroyed and sometimes even disappears in many RA cases. On weight-bearing and hip joint movement, the deformed femoral head continuously abrades the acetabular joint surface. After the biomechanical environment changes and the hip joint center of movement are displaced, the femoral head forces the acetabulum inward to produce an acetabular protrusion. The incidence of such protrusion secondary to hip RA is about 5% and progresses at about 2 mm per year [3]. In some cases, the protrusion does not stop until the femoral neck or proximal greater trochanter is blocked by the margin of the acetabulum [4, 5]. The acetabular protrusion displaces the hip rotational center inwardly, resulting in limb shortening, reduced gluteal muscle tension, and severe hip pain. If the activities of daily living are heavily compromised, total hip arthroplasty is often required. However, total hip replacement in patients with acetabular protrusions developing secondary to RA is very challenging because the severe acetabular alterations increase the difficulty of joint replacement and may also threaten the initial stability of the prosthetic implant [1, 2, 4, 5, 7]. The thin acetabular wall and weak, osteoporotic acetabular loop cannot provide adequate support to the acetabular cup.

Because of the acetabular protrusion, hip movement is significantly limited in all directions. Dislocation of the femoral head from the protrusive acetabulum and exposure of the femoral neck are difficult during surgery [1, 5]; violent dislocation may fracture the acetabular wall [13]. In practice, the femoral head should be excised in a retrograde manner after osteotomy of the femoral neck. However, in patients with severe acetabular protrusions secondary to RA, the femoral head and neck may lie completely within the acetabulum such that even common osteotomy of the femoral neck cannot be performed. We used a grinder or a narrow bone knife to remove the outer parts of the femoral head and neck to increase hip joint abduction; thus, the protrusion of the femoral neck became partially exposed, allowing neck osteotomy. If femoral neck osteotomy remains difficult, additional osteotomy of the greater trochanter should be used to further increase femoral neck exposure. Ultimately, osteotomy of the femoral neck will be complete, safe, and effective; it is easy to control the direction and depth of osteotomy.

When treating protrusions of the acetabulum during total hip arthroplasty for RA patients, the acetabular structure must be carefully considered; the acetabular rim may be weak, the acetabular wall may be thin, and ventral acetabular defects may be present. Bone grafting of the protrusio acetabuli is necessary to restore bone stock, provide a medial buttress for the cup, and adequately lateralize the cup for the restoration of the hip center [10]. A complete bony acetabular rim (creating a hoop) is the ideal support structure for an acetabular cup [14]. It is important to avoid breakage of the acetabular rim during reaming, to ensure rim fixation; because the rim tends to be thin in RA patients. If acetabular rim destruction is extensive, or if dysplasia is evident, an acetabular cage or a cup-reinforcing ring can be used to provide additional mechanical support [1, 3, 5, 12].

The bottom of the acetabulum is always thin and the acetabular wall hardened in acetabular protrusion; the multiple small bleeding subchondral bone holes should be made after the residual cartilage over the thinned medial wall has been removed using curettes or gouges. This allows local blood flow from the pelvic bone, facilitating early bone remodeling and incorporation of grafted bone. The bottom of the acetabulum is filled with the bone graft and compacted, if necessary, using pressure devices [15]; graft consolidation creates a medial buttress for the cup enabling the superomedially directed joint reactive force to be resisted. If structural bone defects are evident in the acetabular wall, the resected femoral head or iliac bone should be trimmed to the appropriate shape and grafted.

Using only cement for acetabular reconstruction in protrusio acetabuli has had unacceptably high rates of recurrence, with components migrating into the acetabulum [16]. Thermal necrosis of the thinned-out medial acetabular wall caused by the heat of polymerization of the cement [17] has been reported to be one of the factors responsible for the poor outcome, especially in protrusio acetabuli secondary to RA patients [18]. In the short term, cemented cups exhibit early loosening, associated with a high revision rate over the first postoperative decade [19–21]. In fact, most patients with protrusio acetabuli secondary to RA are young; cementless acetabular reconstruction is thus very appropriate in such patients, affording significantly better hip survival rates than those of patients with cemented acetabular cups [3, 8, 16]. As the bone bed of the acetabular wall may be of poor quality in such patients, such that extensive granular bone grafting might be required, we prefer to use tantalum trabecular metal cups to reconstruct acetabular protrusions developing secondary to RA. A rougher surface has a higher friction coefficient; a tantalum trabecular metal cup affords excellent fixation and good initial stability. The excellent biocompatibility of trabecular tantalum cups facilitates bone growth [22].

Although we obtained good short-term results, several problems remain. If the femoral head is severely damaged or has disappeared, accompanied by severe acetabular protrusion, a bone graft is sometimes insufficient. We usually place an iliac auto-bone-graft, associated with an additional surgical site. The use of artificial bone is associated with a risk of osteonecrosis, non-union, and collapse. The surgeon can place acetabular cages and rings to afford additional mechanical support when large defects are evident in the acetabular rim or wall, but at the cost of greater surgical complexity and an increased risk of cup malpositioning within the cage or shell. Also, our sample size was relatively small, and we had no long-term follow-up data on cup stability.

## Conclusion

Total hip arthroplasty using impacted morcellized autologous bone and cementless, trabecular, metal modular cups was effective for treating RA patients with protrusio acetabuli. Acetabular reconstruction via bone grafting restores bone stock, creates a medial buttress for the cup, and adequately lateralizes the cup for the restoration of the hip center. However, acetabular structural problems must be carefully considered; possible defects include a weak acetabular rim, a thin acetabular wall, and/or ventral acetabular problems. Although we achieved good short-term results, possible invagination of the acetabular bone graft reconstructions after bone integration, and the long-term stability of acetabular prostheses, requires longer-term follow-up.

## Abbreviations

BMI: Body mass index
THA: Total hip arthroplasty
